# A regional-scale, high resolution dynamical malaria model that accounts for population density, climate and surface hydrology

**DOI:** 10.1186/1475-2875-12-65

**Published:** 2013-02-18

**Authors:** Adrian M Tompkins, Volker Ermert

**Affiliations:** 1 Earth System Physics, Abdus Salam International Centre for Theoretical Physics (ICTP), Strada Costiera 11, Trieste, Italy; 2 Institute of Geophysics and Meteorology, University of Cologne, Kerpener Str. 13, Cologne, Germany

## Abstract

**Background:**

The relative roles of climate variability and population related effects in malaria transmission could be better understood if regional-scale dynamical malaria models could account for these factors.

**Methods:**

A new dynamical community malaria model is introduced that accounts for the temperature and rainfall influences on the parasite and vector life cycles which are finely resolved in order to correctly represent the delay between the rains and the malaria season. The rainfall drives a simple but physically based representation of the surface hydrology. The model accounts for the population density in the calculation of daily biting rates.

**Results:**

Model simulations of entomological inoculation rate and circumsporozoite protein rate compare well to data from field studies from a wide range of locations in West Africa that encompass both seasonal endemic and epidemic fringe areas. A focus on Bobo-Dioulasso shows the ability of the model to represent the differences in transmission rates between rural and peri-urban areas in addition to the seasonality of malaria. Fine spatial resolution regional integrations for Eastern Africa reproduce the malaria atlas project (MAP) spatial distribution of the parasite ratio, and integrations for West and Eastern Africa show that the model grossly reproduces the reduction in parasite ratio as a function of population density observed in a large number of field surveys, although it underestimates malaria prevalence at high densities probably due to the neglect of population migration.

**Conclusions:**

A new dynamical community malaria model is publicly available that accounts for climate and population density to simulate malaria transmission on a regional scale. The model structure facilitates future development to incorporate migration, immunity and interventions.

## Background

Models of malaria transmission are useful tools to aid understanding of the disease dynamic[1] and have long been applied to assess the potential for intervention [2-4]. As weather parameters such as temperature and precipitation are important in determining the disease niche, there is also potential, given accurate forecasts/projections of these parameters, to use malaria models that account for climate in early warning systems in epidemic regions [5,6] or to assess potential shifts in niche regions under climate change scenarios [7], although in both cases incorporation of other socio-economic factors is desirable [8-10].

As in other climate-related sectoral fields, predictive systems are often based on statistical models, which relate past case numbers to climate anomalies through regression techniques [5,11]. The skill of statistical models depends on the data resources available for the region of interest. Such approaches can incorporate interventions, and have increased in complexity to include random errors and relevant socio-economic indicators [12] as in a recent model developed for dengue fever in Brazil [13]. Poor, sparse or short data records confound the derivation of a reliable statistical model.

Dynamical models instead explicitly model the key equations describing the disease dynamic. A good understanding of the disease is required but if this is available this approach has the advantage of being able to incorporate day-to-day and even sub-daily diurnal variations in climate variables and thus differentiate transmission season risk due to sub-seasonal variability [14]. Such models can potentially be applied to different regions to those in which they were developed, although local calibration may still be required.

Early dynamical disease models used idealized equations to describe the vector and host dynamic as separate discrete compartments [2,15]. Many models have focused on the disease development within the host, with rate equations determining the passage between uninfected, infectious and recovering states and vectors given a parasite-free or infectious status. These models frequently include categories to account for immune status of the host, either to improve the model per se [16-18], or to improve our understanding of the process of immunity acquisition by determining which model best fits the available data [19-21]. Often the impact of climate was either neglected or incorporated through the force-of-infection related transmission rates.

Some recently developed dynamical models have focused on improving particular climatic aspects of the vector and parasite life-cycles. A dynamical model for the vector life-cycle has been introduced that also included a treatment of water bodies [22]. The model was designed to be run on a local scale with knowledge of the water body in question, and included the temperature sensitivity of larvae while linking water temperature to air temperature through a function of cloud cover. Another recent study also introduced a simple generic treatment of the rainfall influence on breeding sites into a dynamical model applied to single locations [23]. The Liverpool Malaria Model (LMM), which has been applied regionally to forecasting and climate projection issues [24,25], includes the temperature effect on the sporogonic and gonotrophic cycles, explicitly representing the parasite and egg growth stages, as well as including a treatment of the temperature impact on vector mortality rates[26]. The model was designed to be run over a regional scale but did not include an explicit representation of the surface hydrology; egg laying rates were proportional to the 10-day rain rate. Since flushing of larvae at intense rain rates could be important in reducing malaria [27,28], this relation was modified to a highly nonlinear function to account for this [29,30].

While successfully applied to modelling the parasite-vector cycles, one drawback of these models is that they do not explicitly represent the interaction between host and vector. The lower parasite ratio (*PR*) values in urban areas compared to rural areas is due to a range of socio-economic factors that impact access to health facilities and treatment. Field study and survey based statistical modelling imply a reduction of entomological inoculation rate (*EIR*) with increasing population density in peri-urban environments [31-33], indicating that surface hydrology and decreasing ratio of vector to host is key. Dynamical models that fail to incorporate host-vector dynamics may not differentiate between peri-urban and rural malaria. Moreover, inclusion of the host dynamic allows for an improved representation of human behaviour in malaria propagation, including interventions, treatments and also economic migration, which can reintroduce the disease into malaria-free zones [34-36].

A dynamical model has recently focused on the interaction between the host and vector, which used climate data from a single location in central Tanzania in a set of idealized simulations to investigate the impact of interventions such as bed net usage, although no comparison to data was conducted for the location [37]. Another malaria model [38,39] used an agent-based approach to represent vectors and included population density. The study mapped out a rural village at a 10 m spatial scale, modelling growth of breeding sites using a two dimensional hydrological model and followed the evolution of each individual vector within the village domain. Such a modelling approach would be impossible to apply on a regional scale for planning purposes, due to the shear numerical cost and the lack of the detailed input required on a wide scale. While [38,39] claimed that explicitly modelling each small scale breeding site of the anopheles vector and its interaction with its host was vital to present malaria transmission, we note that in the atmospheric sciences reliable weather predictions are made without explicitly modelling effects occurring below the truncation scale of the model (such as clouds and convection), but instead representing their bulk statistical effects collectively via a so-called *parametrization scheme*[40,41]. In the same way, a regional-scale malaria model can incorporate important small-scale effects such as the growth of available breeding sites (ponds and puddles) in the rainy season, representing the net bulk impact in a parametrization scheme.

The aim here is to introduce a new dynamical malaria model that can be used on a relative fine spatial resolution of order 10 km but applied over a regional scale and explicitly incorporates vector-host interaction in addition to a treatment of the aggregate effect of seasonal temporary bodies in a pond model. While idealized, the hydrological component puts a framework into place that will allow future development such as incorporating land-surface related permanent breeding sites and coupling to a more complex surface hydrology. Moreover, the inclusion of the population density will facilitate the future study of such phenomena as host migration, interventions and urbanization. Thus the model presented here provides a tool that takes a first step towards fulfilling two of the recommendations of [42]: to *understand the relationships between climate and climate-sensitive diseases and health issues under different environmental conditions* by *incorporating other data into... health forecast services, for example population, rural vs. urban residence, [and] migration*. After introducing the model framework, an evaluation the model is conducted in an idealized framework and also using point and gridded regional integrations comparing the model to field studies of entomological parameters, malaria atlas project (MAP) processed survey data and an existing dynamical model. This evaluation demonstrates the ability of model to predict the seasonality and spatial distribution of malaria transmission, before the future directions are summarized.

## Methods

### Overview of the VECTRI model

The malaria model presented is a grid cell distributed dynamical model and is referred to as VECTRI; the *vec*tor-borne disease community model of the International Centre for Theoretical Physics, *Tri*este. This manuscript documents VECTRI release version v1.25 as of June 2012. In as far as possible, the model physics and associated parameters are taken from the literature for the *Anopheles gambiae* complex and the *Plasmodium falciparum* malaria parasite. In the present version each location (grid cell) is independent, but the structure of the model will allow communication between grid cells such as vector flight or human population migration to be easily incorporated. The following sections describe the basic structure of the model, with emphasis placed on its novel aspects.

**Figure 1:**
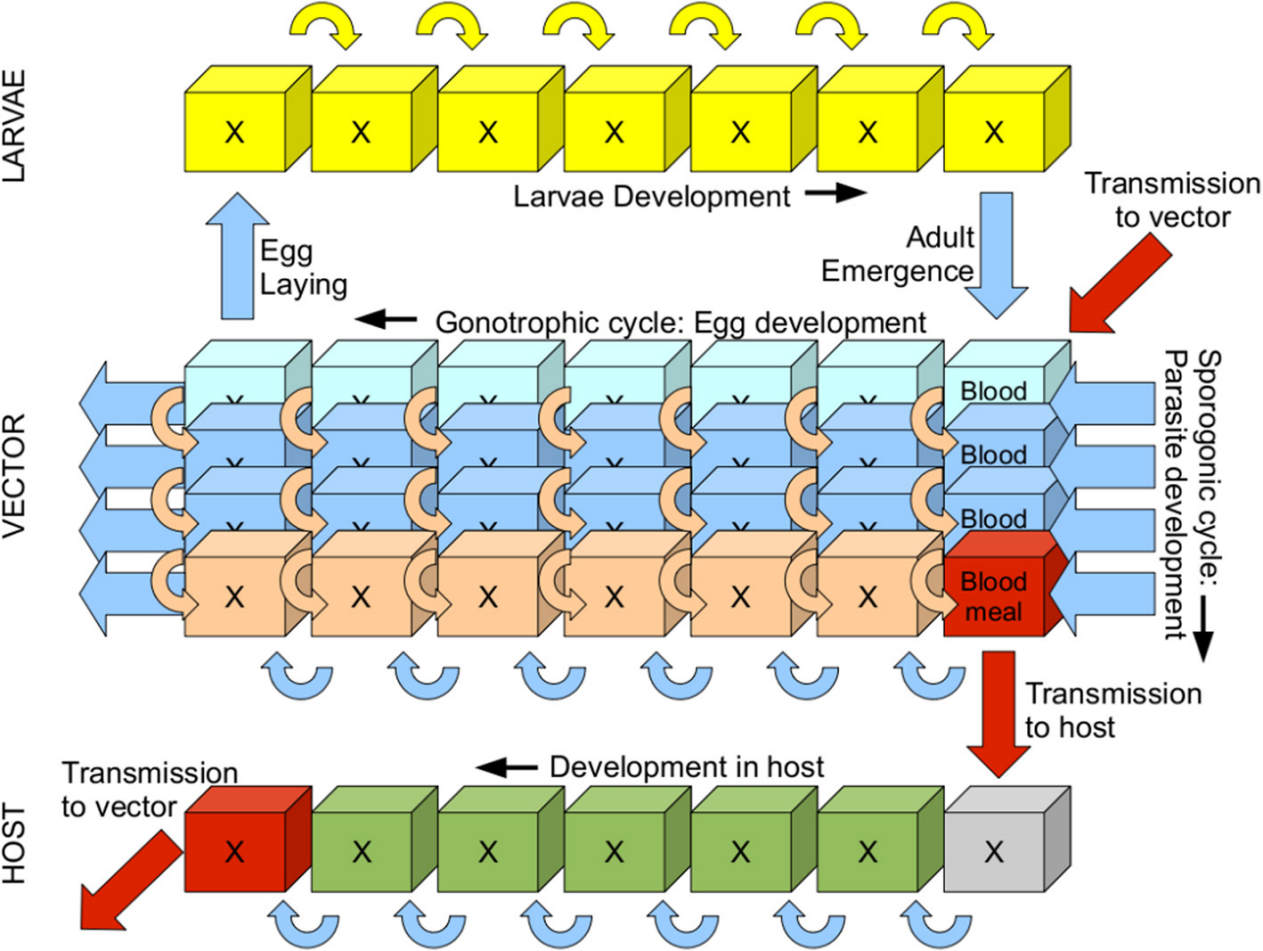
**Schematic of the VECTRI model.** The top row shows the larvae status divided into a series of bins representing the fractional development state. The ’X’ in each bin represents the density of larvae in a specific fractional growth stage. The middle block represents the vector state in two dimensions: the egg development within the female and the infective state. The lower row models the host infective state. The curved arrows represent the progression direction of the larvae, vector and host state, while the straight red arrows mark the parasite transmission pathways between host and vector.

Since one goal of the model is its successful application regionally to forecast epidemic outbreaks in malaria marginal zones in addition to representing malaria transmission in endemic regions, it is important to represent the delay between the rainy season onset and the malaria season. Thus the model explicitly resolves the growth stages of the egg-larvae-pupa cycle in addition to the gonotrophic and the sporogonic cycles using an array of bins for each process, similar to the LMM. The structure of the model is depicted schematically in Figure 1. It shows the division of the larvae life cycle (*L*) into a number (*N*_*L*_) of discrete fractional bins (*i*). The real number stored in each bin, *L*_*i*_, gives the larvae density (per square metre) at a particular fractional growth stage *f* (where *f* ranges from 0 to 1). Oviposition results in eggs added to the first bin and each timestep of the model larvae advance a number of bins at a fractional growth rate *R*_*L*_ (units *s*^-1^), depending on the local water temperature, until they reach the final bin $L_{N_{L}}$ (representing *f* = 1) and develop into adult mosquitoes. The equations solved are thus the classic advection equation:

(1) $$\frac{\text{dL}}{\text{dt}}=R_{L}\frac{\text{dL}}{\text{df}}.$$

The vector status is also bin resolved, consisting of two properties: the gonotrophic and sporogonic cycles. It is thus represented as a two dimensional array *V*(*N*_*gono*_,*N*_*sporo*_). All vectors in the first gonotrophic bin $\overset{N_{\text{sporo}}}{\underset{j=1}{\sum }}V(1,j)$ are in meal-searching mode, and once a meal is obtained, the vectors advance in terms of the egg development state at a rate *R*_*V*,*gono*_ related to ambient temperature until the final bin is reached (using the advection equation similar to eqn. 1). At this point the vector lays a new raft of eggs and is recycled to the first meal-searching bin.

Each timestep, parasite transmission may occur to a proportion of the biting vectors, and the status of these vectors will subsequently additionally progress in the sporogonic dimension, with the rate *R*_*V*,*sporo*_ again determined by temperature. Once vectors reach the final bin they are infective to humans and remain so until death. A third array of bins maps the status of the disease in the human host (*H*) population (dimension *N*_*H*_), with the first bin representing the uninfected population. The model does not include age or immunity factors, which is the subject of present model development.

Thus, while the model introduces new relationships regarding the surface hydrology and the explicit interaction between vector and host, the underlying numerical structure is similar to that employed in the LMM [26]. A timestep of one day is used to integrate the model equations, although a shorter timestep could be used if input data (temperature/rainfall) are available on these timescales, and the advection equation is solved using a simple upstream numerical scheme.

The model can be flexibly integrated using a wide range of horizontal resolutions. Since the model presently does not permit vectors to move to neighbouring grid cells, the model resolution is limited to an upper (finest) level on the order of 1-5 km; an indicated range below which mosquito movement can become significant [43-45]. Integrating the model at O(10 km) resolution is desirable, even if corresponding observational data of clinically proven malaria cases is available only on coarser scale health districts. This is because malaria transmission is a highly nonlinear function of its drivers such as climate and land surface and thus it is preferable to use highest possible spatial resolution inputs of population density, land surface and weather to account for this, while the model output is subsequently aggregated to the spatial scale at which health data is available for comparison.

### Structure of the VECTRI model

The following section describes the details of the parametrizations used to describe the temperature-sensitive progression rates and details the new developments in the VECTRI model concerning the representation of the human population density, which impacts the biting rates, and the treatment of surface hydrology which determines the overall vector number. To enable VECTRI to be used in a multi-model ensemble approach to assess model uncertainties, multiple parametrization choices are incorporated to facilitate this. All the constants are listed in Table 1.

**Table 1:** VECTRI default constants

**Symbol**	**Value**	**Units**	**Description**	**Reference**
*E* + *I*	250	mm day^-1^	Total evaporation and infiltration losses	based on [85]
*K*_*L*,*Jepson*_	90.9	K day	Larvae growth degree days	[47]
*K*_*L*,*Bayon*_	200	K day	Larvae growth degree days	[48]
*K*_*gono*_	37.1	K day	Gonotrophic cycle degree days	[46]
*K*_*sporo*_	111	K day	Sporogonic cycle degree days	[46]
*K*_*flush*,*∞*_	0.4		Larvae flushing factor for infinite rain rate	based on [27]
*K*_*w*_	1	m^-1^	Pond growth rate factor	set by tuning or local knowledge of basin
*K*_*mar*1,0_	0.45		Constant of Martens I vector survival scheme	[61]
*K*_*mar*1,1_	0.054	°C^-1^	Constant of Martens I vector survival scheme	[61]
*K*_*mar*1,2_	-0.0016	°C^-2^	Constant of Martens I vector survival scheme	[61]
*K*_*mar*2,0_	-4.4		Constant of Martens II vector survival scheme	[62]
*K*_*mar*2,1_	1.31	°C^-1^	Constant of Martens II vector survival scheme	[62]
*K*_*mar*2,2_	-0.03	°C^-2^	Constant of Martens II vector survival scheme	[62]
*M*_*L*,*max*_	300	mg m^-2^	Carrying capacity of water bodies	[38]
*N*_*egg*_	120		Number of eggs per batch that result in female vectors	[30]
*P*_*L*,*surv*__0_	0.825		Larvae base daily survival rate	[29]
*P*_*hv*_	0.2		Probability of transmission from infective host to vector during single bloodmeal	[29]
*P*_*vh*_	0.3		Probability of transmission from infective vector to host during single bloodmeal	[29]
*T*_*wat*_	2	K	Pond water offset from air temperature	[92]
*T*_*L*,*min*_	16	°C	Minimum *T*_*wat*_ for larvae development	based on [48]
*T*_*L*,*max*_	38	°C	Maximum *T*_*wat*_ for larvae development	based on [48]
*T*_*gono*,*min*_	7.7	°C	Minimum *T*_2*m*_ for egg development	[46]
*T*_*sporo*,*min*_	16	°C	Minimum *T*_2*m*_ for sporogonic cycle	[29]
*τ*_*flush*_	50	mm day^-1^	Larvae-flushing rainfall e-folding factor	based on [27]
*τ*_*zoo*_	50	km^-2^	Population density zoophilic factor	set by tuning
*w*_*max*_	0.04		Maximum temporary pond fraction in cell	set by tuning or local knowledge of basin

List of constants in VECTRI.

#### Larvae cycle

Previous laboratory studies have shown that the larvae growth rate follows the degree day concept [46] based on a linear function of water temperature *T*_*wat*_ above a threshold value *T*_*L*,*min*_ below which larvae growth ceases:

(2) $$R_{L}=\frac{T_{\text{wat}}-T_{L,\text{min}}}{K_{L}}.$$

There is considerable uncertainty in the setting of the rate coefficient *K*_*L*_, however, with [47]*K*_*L*_ value of 90.9 degree days, while a linear approximation of the relationship derived by [48] results in a much slower rate of 200 degree days. A further source of uncertainty is the specification of the water temperature itself, which depends on the shading of the pool and its dimensions in addition to the ambient air temperature, and is described in the hydrology component below. VECTRI also permits the user to avoid this uncertainty by following [29] in setting a fixed larvae growth rate (i.e. independent of *T*_*wat*_) to have a cycle of 12 days. The sensitivity to this relationship is investigated later. Irrespective of the scheme used, an upper temperature limit *T*_*L*,*max*_ is specified above which larvae death occurs.

Egg hatching into larvae and the pupae development stage are both typically on the order of one day [48,49] and thus are poorly resolved by the daily timestep employed by VECTRI and other similar dynamical models. In order to avoid truncation problems the length is fixed in VECTRI to last exactly one day and is temperature independent.

#### Larvae mortality

The mortality rate of larvae is an important factor for transmission, and is strongly temperature dependent [50]. The VECTRI model sets a base daily survival rate for larvae *P*_*L*,*surv*__0_ = 0.825 taken from [29], slightly lower than the values given by [26,38]. Development of larvae is negatively affected by larvae over-population due to competition for resources [51]. This is incorporated in VECTRI by reducing survival rate proportionally by a factor related to resource constraints:

(3) $$P_{L,\text{surv}}=\left(1-\frac{M_{L}}{wM_{L,\text{max}}}\right)K_{\text{flush}}{P_{L,\text{surv}}}_{0}.$$

In the first term on the right, *M*_*L*_ is the total larvae biomass per unit surface area of a water body, and *w* is the fraction coverage of a grid cell by potential breeding sites (not open water) and is given by the surface hydrology component described below. If *w* = 0, the survival rate *P*_*L*,*surv*_ is also zero. The maximum carrying capacity, *M*_*L*,*max*_, is set to 300 mg m^-2^ and larvae mass is assumed to increase linearly with a stage 4 larva having a mean mass of 0.45 mg, both following [22,38] closely. All larvae die above a water temperature of *T*_*L*,*max*_.

**Figure 2:**
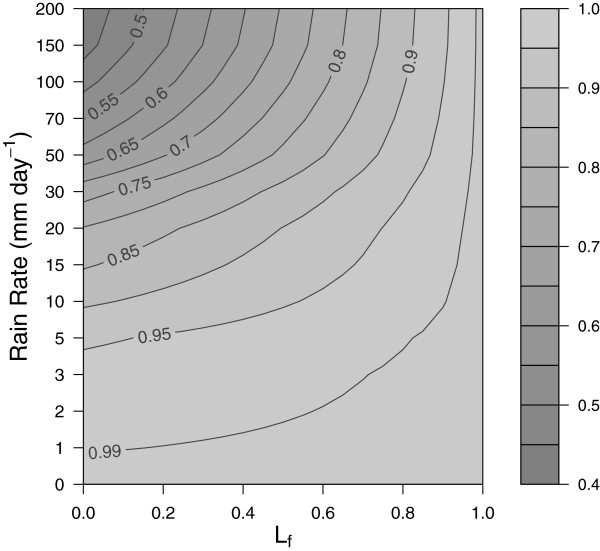
**Larvae Flushing.** Fractional reduction in larvae survival rate (*K*_*flush*_) as a function of larvae growth stage (*L*_*f*_) and rain rate as implemented in VECTRI.

Flushing of larvae by heavy rainfall has been suggested to be an important cause of larvae mortality [52,53]. Using an artificial pond apparatus [27], a 17.5% daily mortality rate of first stage larvae reducing to 4.8% for forth stage larvae was estimated, although the authors state that these figures could represent an underestimate due to the symmetry of the pond apparatus and the lack of sampling of more extreme rainfall amounts during the experiment campaign. On the other hand, vegetation in natural pools and the apparent ability for *Anopheles gambiae* larvae to take avoidance measures to avoid flushing could imply a lower flushing rate [54]. In order take the simplest possible relationship in VECTRI, flushing rate is represented as exponential function of rain rate, and is related linearly to the larvae fractional growth state *L*_*f*_:

(4) $$K_{\text{flush}}=L_{f}+(1-L_{f})\left((1-K_{\text{flush},\infty })e^{\frac{-R_{d}}{\tau_{\text{flush}}}}+K_{\text{flush},\infty }\right),$$

where *R*_*d*_ is the rainfall rate in mm day^-1^, *τ*_*flush*_ describes how quickly the effect increases as a function of *R*_*d*_, and *K*_*flush*,*∞*_ is the maximum value of *K*_*flush*_ for newly hatched first stage larvae at extremely high rain rates. In contrast the flushing effect is zero (*K*_*flush*_ = 1) at all rain rates for stage 4 larvae just prior to adult emergence (*L*_*f*_ = 1, corresponding to the larvae bin *i* = *N*_*L*_). The relationship is illustrated in Figure 2. Since flushing is a function of daily rain rate at relatively high spatial resolution *τ*_*flush*_ is set to 50 *τ*_*flush*_ is set to 50 mm day^-1^. In order to give mortality rates of first stage and forth stage comparable to [27] for typical daily rain rates in the tropics, a value of 0.4 is adopted for *K*_*flush*,*∞*_.

#### Gonotrophic cycle

All female vectors are assumed to find a blood meal in the first night of searching, although this fraction can be set as a model parameter. Insecticide treated nets are, for example, able to frustrate host-seeking mosquitoes [55] and reduce the mosquito population number. It would depend on the human population and availability of animals. In this way both newly emerging and existing adult mosquitoes might be prevented from progressing in the feeding cycle.

Once the blood meal is taken, the egg development proceeds at a rate determined by the local 2 metre air temperature *T*_2*m*_, again following the degree day concept and is thus given:

(5) $$R_{\text{gono}}=\frac{T_{2m}-T_{\text{gono},\text{min}}}{K_{\text{gono}}}.$$

At the end of the cycle the female vector lays *N*_*egg*_ eggs that will eventually hatch into females; as is usual in such models, the eggs laid that result in the males are neglected. She subsequently cycles to the meal searching box. The number of eggs is highly variable and depends on vector species. The choice of *N*_*egg*_ = 120, corresponding to a batch size of 240 assuming equality between the sexes, follows [30] but [56-58] indicate that this could be an overestimation. Present model development underway will permit stochastic variation of parameters such as *N*_*egg*_ in the future to account for their considerable degree of uncertainty in an ensemble modelling framework.

#### Sporogonic cycle

When the female vector takes a blood meal there is a certain finite probability that malaria transmission takes place, either from the host to vector or vice versa. The probability of transmission to the vector during a blood meal from an infective host is considered a constant *P*_*hv*_ = 0.2 (following [29]), and thus the overall transmission probability *P*_*h* → *v*_ is the product of *P*_*hv*_ and the proportion of hosts that are infective, namely the ratio of the population density of infected hosts *H*_*inf*_ divided by the population density *H*:

(6) $$P_{h\to v}=\frac{H_{\text{inf}}}{H}P_{\text{hv}}.$$

It should be noted that this assumes bites are randomly taken and not influenced by the host’s infective state. Heterogeneous biting can impact the basic reproductive number considerably [59,60]. Heterogeneity of feeding habits is related to a wide array of factors, including host attractiveness to the vector and their vicinity to breeding sites, and heterogeneity in interventions such as the use of bed nets, in particular the increased use by hosts suffering from clinical malaria. Some of these effects could easily be included in VECTRI if relevant data were available.

Each timestep, a proportion of vectors *P*_*h* → *v*_ become infected, and the parasitic development in the midgut of the vector begins, once again governed by a degree day concept:

(7) $$R_{\text{sporo}}=\frac{T_{2m}-T_{\text{sporo},\text{min}}}{K_{\text{sporo}}}.$$

After a number of days the sporozoites invade the salivary glands of the mosquito which subsequently becomes infective to humans. The mosquito is assumed to remain in this infective state until its death.

#### Vector survival

In addition to the larvae, gonotrophic and sporogonic cycles, temperature also plays a role in determining the mortality of vector. High air temperatures increase vector mortality, but the relationship is uncertain, especially at the high and low temperature bounds of transmission. As in the larvae cycle, two schemes are incorporated in the VECTRI model to permit a multi-model approach, with constants given in Table 1:

Scheme 1: follows [52,61] and gives the survival rate as a quadratic function of temperature:

(8) $$P_{V,\text{surv}1}=K_{\text{mar}1,0}+K_{\text{mar}1,1}T_{2m}+K_{\text{mar}1,2}T_{2m}^{2}$$

Scheme 2: follows [62,63], revising the relationship as

(9) $$P_{V,\text{surv}2}=\text{exp}\left(\frac{-1.0}{K_{\text{mar}2,0}+K_{\text{mar}2,1}T_{2m}+K_{\text{mar}2,2}T_{2m}^{2}}\right)$$

The results in this paper are obtained using scheme 2, referred to in [29] as *Martens II*.

### Host community

One of the new aspects of the VECTRI model is that it explicitly allows the interaction between vector and host population on a district and regional scale. The VECTRI model specifies the population density *H* using the Africa-only AFRIPOP [64] or global GRUMP [65] datasets which have a nominal 1 km and 4.5 km spatial resolution, respectively. Thus at each location (model spatial grid cell), the ratio of biting vectors to hosts is known and is given by ($\overset{N_{\text{sporo}}}{\underset{j=1}{\sum }}V(1,j)/H$). This is important to represent the vector-to-host transmission rate. The number of bites *B* that any particular individual receives in a given time the human biting rate (*hbr*) is considered to be a random process, and thus distributed following a Poisson process with a mean biting rate of

(10) $$\bar{\text{hbr}}=\left(1-e^{\frac{-H}{\tau_{\text{zoo}}}}\right)\frac{\overset{N_{\text{sporo}}}{\underset{j=1}{\sum }}V(1,j)}{H}.$$

The factor $1-e^{\frac{-H}{\tau_{\text{zoo}}}}$ represents the level of vector zoophily. While members of the *Anopheles gambiae* complex are in general considered anthropophilic to varying degrees [66], with *arabiensis* more zoophilic than *sensu stricto*[67], vectors take an increasing proportion of blood meals from cattle in lower population density rural areas with high livestock numbers [68], although the effectiveness of zooprophylaxis is still debated [69]. The exponential factor reflects this, with the e-folding population density for the effect set to *τ*_*zoo*_ = 50 km^-2^. Thus the factor only has a significant impact for rural populations below this number and avoids the model producing excessively high biting rates and *EIR* for sparsely populated locations. In future, VECTRI will allow vector movement between cells allowing anthropophilic vectors to cluster around population centres.

The daily number of infectious bites by infectious vectors, *EIR*_*d*_, is the product of *hbr* and the circumsporozoite protein rate (*CSPR*). Specifically in the VECTRI notation, this is *V*(1, *N*_*sporo*_) / *H*, with the 1 indicating that the calculation is restricted to the vectors that are biting within the present timestep of the model. This implicitly assumes that there is no change in the intensity of biting or the gonotrophic cycle length between uninfected and infectious vectors; a simplification according to [70]. If the transmission probability from vector to host for a single bite of an infective vector, *P*_*vh*_, is assumed a constant then the transmission probability for an individual receiving *n* infectious bites will be 1 - (1 - *P*_*vh*_)^*n*^. The impact on transmission due to blocking immunity is neglected. Thus the overall transmission probability per person per day in the model can be obtained by the integration over the bite distribution:

(11) $$P_{v\to h}=\overset{\infty }{\underset{n=1}{\sum }}G_{\bar{\text{EI}R_{d}}}(n)\left(1-{\left(1-P_{\text{vh}}\right)}^{n}\right)$$

where $G_{\bar{\text{EI}R_{d}}}$ is the Poisson distribution for mean $\bar{\text{EI}R_{d}}$. If bed nets are in use, eqn. 11 could be modified to incorporate this, increasing the mean bite rate for a subset of the unprotected population. This involves a number of complications however, since accurate data would be required concerning bed net distribution and use, how this usage correlates to host infective state, and which proportion of bite are taken during the hours of sleeping.

**Figure 3:**
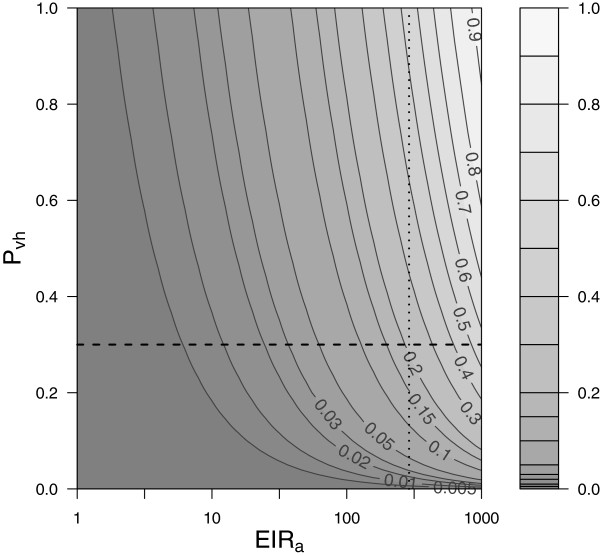
**Vector to host transmission.** Contour plot of *P*_*v*→*h*_ as a function of *P*_*vh*_ and *EIR*_*a*_. The horizontal dashed line highlights *P*_*vh*_ = 0.3 as specified by the default VECTRI model. The vertical line marks *EIR*_*a*_ = 365, for which *P*_*v*→*h*_ would equal *P*_*vh*_ if the distribution in biting rate were not taken into account.

The impact of using eqn. 11 is to reduce the mean transmission rate, particularly when the mean bite rate is small, resulting in a strong positive skewness of the Poisson distribution (Figure 3). While this is an improvement on the simple assumption that all hosts receive equal numbers of bites, the Poisson distribution is likely under-dispersive compared to reality, since a number of factors such as unequal host attractiveness to vectors and nocturnal behaviour affecting exposure will likely lead to an uneven distribution of biting rates [71-73]. Figure 3 also emphasizes that the model is relatively insensitive to the choice of *P*_*vh*_ for values exceeding around 0.2. The default value that VECTRI adopts for *P*_*vh*_ is 0.3 following [29], but there is considerable uncertainty in this parameter, which also depends on immune status, with [74] reporting that half of a small group of 10 malaria naive volunteers were infected after being exposed to 1 to 2 infective bites, while [75] estimated a value of just 1 in 13 in an endemic area.

The host population is represented by the vector *H*(*N*_*host*_), and each VECTRI timestep a proportion *P*_*v* → *h*_ of hosts become infected and progress through the array until 20 days later they assume an infective status, an average value for immune and non-immune subjects [76-79]. Non-immune hosts clear infections at an e-folding rate of *C*_*ni*_ = 150 days. Even after a century of study of the disease, the paradigm of naturally acquired immunity (NAI) is still hotly debated [21]. Therefore the present version of the model neglects host immunity, and the impact of the various representations of immunity in VECTRI will be the subject of a companion article.

### Surface hydrology

The VECTRI model includes a simple, physically-based surface hydrology model that at each timestep provides a calculation for the fractional coverage of each model gridcell by potential breeding sites for the malaria vector, *w*. This fraction consists of two components since the model distinguishes between breeding sites provided by temporary ponds, *w*_*pond*_, and those associated with permanent water bodies such as lakes, rivers and streams that contain water year-round, *w*_*perm*_:

(12) $$w=w_{\text{perm}}+w_{\text{pond}}.$$

Considering first *w*_*perm*_, converting land use and terrain information into a fractional coverage of breeding areas provided by permanent water bodies is a significant challenge. Wave/ripples action that can drown larvae [80] and the presence of predators in larger bodies [81] imply that larvae exist only in a sub-fraction of such water bodies, in pooling that occurs on the edges of lakes and rivers or the shallow edges of ponds. Higher soil moisture in the vicinity of large water bodies can boost available breeding sites by reducing infiltration loss and increasing the lifetime of temporary puddles and ponds. Lastly, rivers and streams can even confound classic relationships between rainfall and vector density by actually providing more breeding sites during drought periods when flow slows or stops altogether [82]. This remains the subject of current research and the default VECTRI model therefore sets *w*_*perm*_ to zero in each grid cell, implying that the breeding site availability is dominated by seasonal ephemeral ponds and malaria incidence will be potentially underestimated in the vicinity of larger semi-permanent water bodies. A user operating the model for a local area with knowledge of permanent water bodies can set this value appropriately.

The net aggregated fractional coverage by temporary pools *w*_*pond*_ is derived from a simple water balance model. Ponds are replenished by surface runoff *Q*, while infiltration *I* (seepage) and evaporation *E* and pond overflow reduce their water content. An important parameter is the maximum coverage of the temporary ponds *w*_*max*_, which described the extent of depressions that could potentially become water filled at the peak of a wet season, which are often small in scale, with total catchments of the gully systems studies in Niger in the HAPEX-SAHEL experiment measuring 0.2 km^-2^[83]. Presently it is assumed that the runoff *Q* that fills the ponds mostly falls within these depressions and thus the runoff is set to *Q* = *w*_*max*_*P*, where *P* is the precipitation rate. Thus sub-surface infiltration occurring within the depressions is also considered a water source for temporary pools [83]. Future developments will introduce an improved runoff treatment accounting for soil texture and slope.

The source from precipitation is balanced by evaporative, infiltration and overflow losses. In high-resolution simulations of surface hydrology in Niger, [84] found overflow losses to be approximately 20% of total losses, more than three times the losses due to evaporation. It should also be noted that overflow losses in field campaigns are difficult to measure and thus are often incorporated in the infiltration, which is calculated as a residual in the water balance calculation. Losses through pond overflow are assumed to increase linearly with pond fraction in VECTRI, achieved by scaling the runoff by a factor $1-\frac{w_{\text{pond}}}{w_{\text{max}}}$. Once the pond fraction reaches its maximum, all surface runoff overflows and is lost. Infiltration losses vary substantially depending on soil texture and life-scale of the pond in question. Often the infiltration is a highly nonlinear function of water body extent, since silting may significantly reduce infiltration in the lowest part of longer-lived or semi-permanent pools [83]. This results in a fast initial decay after rain events due to high infiltration rates at the pool edges, followed by a slower decay, while temporary, shorter lived water bodies tend to have more uniform infiltration rates. These rates can be very high, exceeding 600 mm day^-1^[85]. Presently the VECTRI model simply sets a fixed constant infiltration rate per unit pond area.

Combining these factors, the volume of water *v*_*pond*_ in ponds per unit area thus evolves as

(13) $$\frac{dv_{\text{pond}}}{\text{dt}}=w_{\text{max}}P\left(1-\frac{w_{\text{pond}}}{w_{\text{max}}}\right)-w_{\text{pond}}(E+I).$$

Evaporation is set to 5 mm day^-1^, equivalent to a latent heat flux of 145 Wm^-2^. It is possible to derive evaporation losses from water temperature, and atmospheric wind speed and relative humidity, however, as evaporation is a relative minor loss term relative to infiltration and overflow, a simple fixed evaporation rate suffices. With *I* set to a reasonable value of 245 mm day^-1^, total losses day^-1^, total losses from infiltration and evaporation are thus 250 mm day^-1^. For closure, the pond fractional coverage needs to be related to the volume. Individual ponds have been modelled previously using a power law approximation [86], which would lead to a relationship $\frac{\text{dw}}{\text{dt}}\propto w^{-p/2}\frac{\text{dv}}{\text{dt}}$, where *p* is the power law exponent. However, [86,87] show that *p* can vary by almost an order of magnitude from one water body to another and depends in particular on the lifetime. In the present version model this factor is neglected and the coverage is simply linearly related to pond coverage introducing a tunable factor *K*_*w*_:

(14) $$\frac{dw_{\text{pond}}}{\text{dt}}=K_{w}\left(P(w_{\text{max}}-w_{\text{pond}})-w_{\text{pond}}(E+I)\right).$$

**Figure 4:**
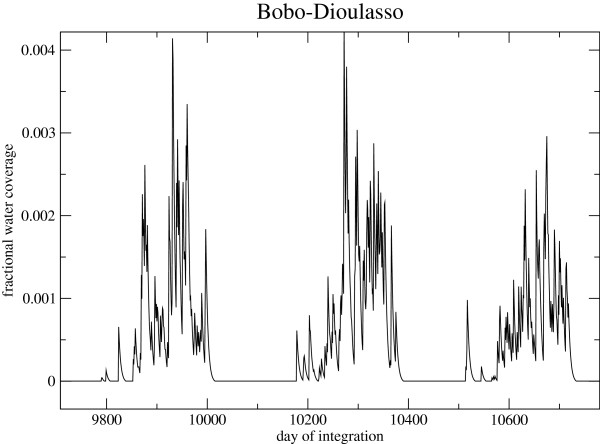
**Surface hydrology.** An subsection of a multi-year VECTRI integration for Bobo-Dioulasso showing the evolution of model water fraction for three rainy seasons.

As the pond coverage can change rapidly, eqn. 14 is integrated using a fully implicit solution. An example evolution of the fractional pond coverage for a site near Bobo-Dioulasso is given in Figure 4, which shows that the simple empirical approach mimics the pond evolution modelled by high resolution hydrological models for sites in Niger [38] and ponds modelled in Senegal [88]. The present empirical formulation is similar to the approaches of [23,37]. It is seen that at the fringes of the rainy season, puddles and small ponds have limited longevity on the order of a few days, implying that they are unsuitable for vector breeding. VECTRI represents the bulk behaviour of ponds, rather than the ultra-high resolution model of [84] which individual models puddles at the 10 m scale and thus can model the lifetime of ponds as a function of their explicit size (see their Figure Seven). Obviously, the linear relation between rainfall and the growth of potential breeding sites in parameter *K*_*w*_ is a simplification, while the other terms should be related to atmospheric conditions, soil type, vegetation coverage and terrain slope demonstrated to be important for malaria transmission in the Kenyan highlands [89].

**Figure 5:**
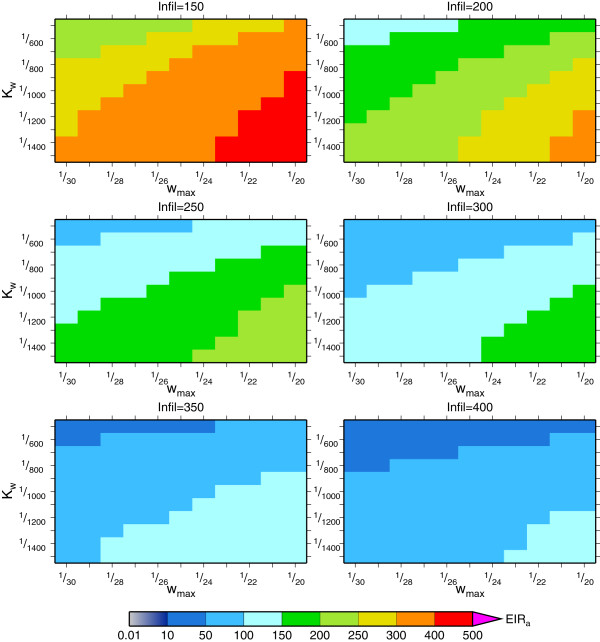
**Sensitivity of pond model to parameter settings.** Simulated mean *EIR* for multi-year VECTRI integrations at Bobo-Dioulasso between 1973 and 2006 with each pixel giving the value for an parameter setting according to certain *w*_*max*_ and *K*_*w*_ values, with each panel representing a different infiltration rate.

Presently, the two unknowns *w*_*max*_ and *K*_*w*_ in the framework are set using a simple Monte Carlo suite of station data integrations in a subset of locations to minimize *EIR* errors compared to field data. An example sensitivity integration is shown in Figure 5, which is a integration conducted for Bobo-Dioulasso using station data to drive the model (see below for experimental set up details). It is seen that transmission intensity increases with *w*_*max*_ and *K*_*w*_ as expected, since these increase the pond coverage for a given rain rate, while increasing the loss rate acts in the opposite direction. For a given station there are a range of reasonable parameter values, with the present parameter settings chosen using a small number of locations in West Africa. Nevertheless, the physically based framework facilitates future improvement currently underway, which will include direct validation of the revised hydrological model constants using in situ and remotely sensed data.

Finally, it is recalled that the pond dimension limits larvae mortality rates through the availability of breeding sites governed in eqn. 3. This is an approximation of the net affect of crowding which leads to higher mortality rates, longer development times and smaller adults [90], which in turn have a competitive disadvantage [58]. The biomass is considered to be distributed equally through all available breeding sites and variability between breeding sites in neglected, supported by [91] who noted that females avoid ponds that are overcrowded with existing larvae.

In addition to pond dimension the other important parameter of water bodies is the temperature of the water near the surface. The Depinay model [22] developed a complex empirical function for water temperature as a function of ambient relative humidity and water body size. As the VECTRI model is applied regionally, specific information about individual water body size may not be included. The temperature in shallow ponds and puddles is homogeneous to a good approximation and is often one or two degrees warmer than the air temperature [92,93]. VECTRI therefore assumes that the temperature of pools *T*_*wat*_ to have a fixed offset relative to the air temperature. The default value adopted is a positive offset of 2 K, however, in hot locations it is likely that vector will preferentially choose shaded breeding locations and a lower or even negative offset may be more appropriate. If accurate gridded weather information for wind and surface radiation were available, this aspect of the model could be potential improved implementing a single energy balance model along the lines of [94,95]. While larger permanent water bodies such as lakes and rivers can have complex stratification of the vertical temperature profile, as discussed above, larvae development occurs mostly in the shallow waters and pools that form on the lake/river boundaries and thus the temperature relation for the permanent water fraction is treated in the same way as the temporary ponds.

### Evaluation of VECTRI

#### Evaluation methodology and data

VECTRI is evaluated using a series of three testbed arrangements. The first test determines the sensitivity of the force of infection measured by *EIR* in relation to climate in an idealized setting of so-called ’equilibrium runs’. The model is driven to equilibrium in an array of experiments using constant values of temperature and rainfall and compared to the reference model described below.

The VECTRI model is next confronted with a wide range of entomological data consisting of *EIR* and circumsporozoite protein rate (*CSPR*) measurements collected from field campaigns located throughout West and Central Africa and which were collated in a review by [96]. These campaigns are divided between rural and urban environments. In particular, the availability of a large number of both peri-urban and rural field studies in and nearby Bobo-Dioulasso documented in [97-106] lead to this location being used as a focus site. For each location, the models are driven by daily weather data from the nearest available station, which for points in or nearby population centres is often within 10 km, but can be much further for isolated rural locations. A simple interpolation methodology is used to fill temporal data gaps [96]. While the malaria field campaigns are often only available for a single year, and never more than a few seasons, the malaria models are integrated for over four decades to give an indication of interannual variability, with results from these ’station integrations’ shown from 1973 to 2006.

Lastly, the malaria prevalence is evaluated from ’regional integrations’ conducted with VECTRI, the first in Eastern Africa (domain 27°E-42°E and 12°S-6°N) and the second for West Africa (18°W-9°E and 4.5°N to 19.5°N). Integrations last for 10 years and use a horizontal spatial resolution of 0.1 degrees, equivalent to approximately 11 km. As freely available daily station data is sparse, the model is instead driven with satellite-derived rainfall using the NOAA FEWS RFE product [107], available from late 2000 to the present on a 11 km resolution which is interpolated to the VECTRI grid using conservative remapping. The gridded 2 metre height temperature data is taken from the ECMWF ERA-Interim reanalysis product [108]. As the resolution of the reanalysis is relatively coarse at approximately 75 km in the tropics, it is statistically downscaled using a constant adiabatic lapse rate of 6.5 K km^-1^[109] to correct for the height error between the model topography and the ETOPOv2 topography [110] bilinearly interpolated to the VECTRI grid. Starting from artificial initial conditions of 5% of the population carrying the parasite, the model is integrated for a spin up period of 3 years cycling the first year of weather data to allow the malaria loading in the population to equilibrate before the integration runs freely. Focus is on the integration for Eastern Africa due to the wide range of topography and the consequential inclusion of malaria free, epidemic and endemic zones, which are compared to the MAP malaria analysis [111-113]. The MAP analysis ingests *PR* survey data occurring up to 2010 into a statistical model that also incorporates environmental data to provided gridded values of *PR*[114]. The reliability of the MAP data depends on the local availability of survey data. In Africa, for most areas there is a large uncertainty of the MAP prediction. For example, the prediction is less certain for Uganda and Tanzania, which represent under-surveyed countries. More certain is the MAP analysis for most parts of Kenya, although [113] marks the coastal and western regions with high uncertainty despite the far greater densities of survey data available there. The two regional runs are thus also compared to direct survey data from 199 field campaigns using the analysis of [33] in order to assess the impact of population density on *EIR*.

#### Comparison model

The VECTRI model is contrasted to the Liverpool Malaria Model (LMM), which has been applied in various forms to regional malaria modelling across Africa for both seasonal timescale forecasting [24] and to investigate climate change impacts on transmission [115]. The original version of LMM described by [26] was updated in terms of its surface hydrology by [29,30], and referred to here as LMM_2010_. The LMM_2010_ is a dynamical mathematical-biological malaria model, which is driven by the two variables of daily mean temperature and precipitation. The treatment of surface hydrology in LMM_2010_ differs significantly to VECTRI. It applies a fuzzy distribution model to relate egg-larvae development to the decadal (10 day accumulated) rainfall amount, with optimal conditions assumed to occur with 1 mm day^-1^ average rain rate. rain rate. Egg-laying rates are reduced below 1 mm day^-1^ rain rates to reflect the lack of breeding sites, and also above this threshold since flushing effects are then assumed to dominate. The use of a ten day average rainfall essentially equates to the assumption that rainfall feeds ponds and pools which have a ten day mean lifespan. Water temperatures are not considered by the LMM_2010_ in the egg-larval development rates.

The LMM_2010_ uses similar entomological and parasitological numerical components to VECTRI. By contrast, the LMM_2010_ neglects the population density and is only calibrated to malaria observations from rural locations in West Africa. This means that the LMM_2010_ is not able to simulate urban malaria conditions. The model is also not valid for irrigated malaria areas, where malaria transmission is not depending solely on rainfall. The vector-host contact is furthermore static in the LMM_2010_ since a fixed proportion of the mosquito bites is taken up by humans and animals.

## Results and discussion

### Equilibrium integrations

**Figure 6:**
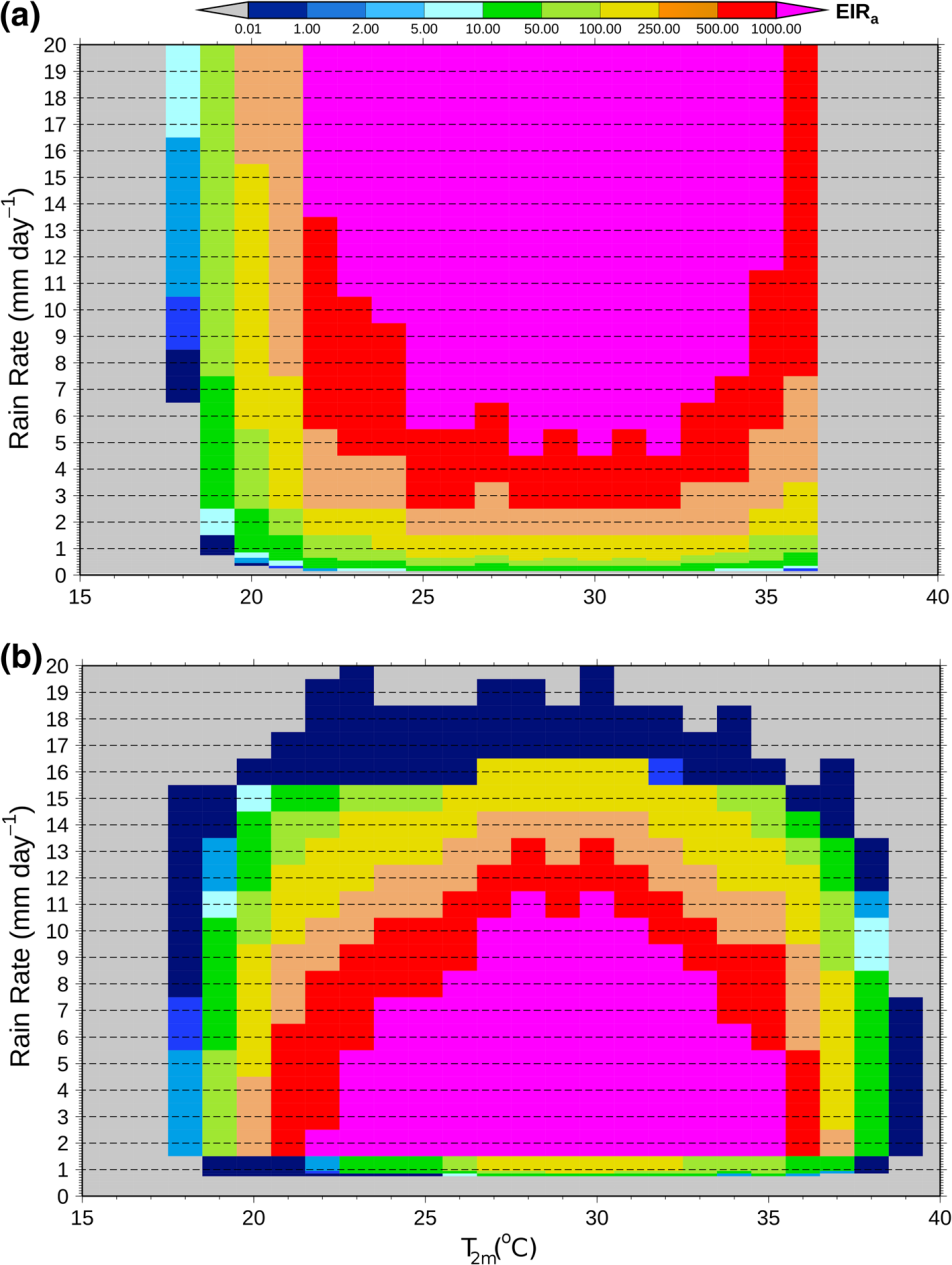
**Equilibrium integrations.** Equilibrium integrations of (**a**) VECTRI and (**b**) LMM_2010_ as a function of constant input of daily mean temperature (*T*_2*m*_) and daily rainfall amounts.

The VECTRI model is driven to equilibrium in an array of experiments using constant values of temperature and rainfall, and compared to the reference LMM_2010_ (Figure 6). In terms of temperature, the range at which malaria transmission occurs (*EIR*>0.01) of 18 to 37 degrees is similar to the LMM_2010_, although the upper limit is controlled by the maximum in water temperature and thus is lower than the LMM_2010_, which ignores the impact of water temperature. It is noted that, unlike larvae which have limited potential to avoid high water temperatures, mosquitoes are able to change behaviour such as resting habits to avoid extreme air temperatures [116]. Therefore, the usual attribution of the upper temperature limit of malaria transmission to excessive vector death, as also determined by the LMM_2010_, may be misleading. On the other hand, the water temperature is an uncertain parameter, and greatly variable shading and availability of sites in a grid cell may imply the water temperature is a less important parameter than indicated by VECTRI.

In terms of rainfall sensitivity, the two models differ considerably. The nonlinear function that determines number of egg laid as a function of rainfall in the LMM_2010_ model is very sensitive to rainfall. As the rain rate deviates from the assessed ideal value of 1 mm/day, the *EIR* decreases quickly. Thus in the rainy season, when daily rainfall amounts usually exceed 1 mm/day, it is likely that the LMM will produce an anticorrelation between rainfall amount and malaria transmission intensity. Instead, although the VECTRI model includes a flushing parametrization, the effect is much weaker at these moderate rain rates of 0 to 20 mm day^-1^ and rainfall is therefore positively correlated with transmission transmission intensity. Moreover, above 2 to 3 mm day^-1^, the sensitivity of *EIR* to rain rate is much lower in VECTRI than LMM_2010_, which is likely to result in a lower interannual variability of malaria in endemic regions in the former model.

### Station integrations

**Figure 7:**
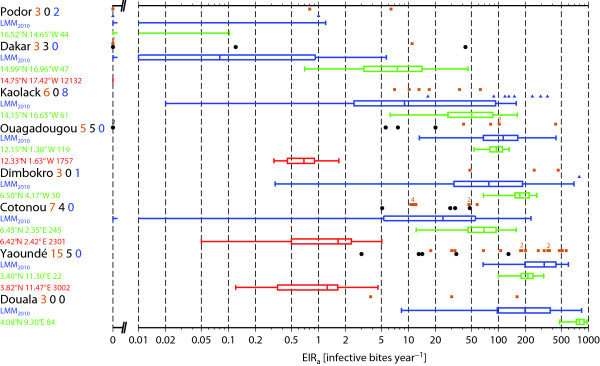
**Observations and simulations of *EIR *and *CSPR*.** Box-and-whisker plots of the VECTRI and LMM_2010_ simulated annual Entomological Inoculation Rate (*EIR*_*a*_; in infectious bites per human per year) for eight West African locations. The box-and-whisker plots of the LMM_2010_ are illustrated in blue. VECTRI results in terms of rural and urban areas are taken green and red, respectively. Also the latitudinal, longitudinal position, and used population density (km^-2^) is indicated. Climate varies from a semi-arid (Podor) to a tropical climate (Douala). Field *EIR*_*a*_ observations are furthermore included as brown squares, black dots and blue triangles in rural, urban and irrigated areas, respectively. The number of field observations is entered behind the location names in the following order: 1) rural, 2) urban, and 3) irrigated observations. The number of clustered observations on the logarithmic scale is indicated by a digit above the symbols.

A summary of the results for a selection of stations across the region (Figure 7) the control model LMM_2010_ agrees well with the range of *EIR* values, which is not surprising, considering these data were used to calibrate the model settings. Although VECTRI was not calibrated with the data, at seven of the eight locations the rural VECTRI *EIR*_*a*_ values overlap with the field observations meaning that the VECTRI runs produce realistic transmission values for most parts of West Africa. In comparison to the LMM_2010_, VECTRI shows a much smaller year-to-year variability and some field observations lie outside the range of the model interannual variability. To a certain extent, this is to be expected, since the model is only able to simulate the interannual variability due to climate - other factors such as interventions are neglected. Instead, the LMM is calibrated to reflect all variability in the observations in its sensitivity to rainfall and temperature, as seen in the earlier equilibrium integrations. That said, VECTRI underestimates the malaria transmission in the northern Sahel. Too small transmission values are simulated by VECTRI in Podor for example. Transmission in urban areas appears to be under-simulated, and in two urban locations in Dakar no transmission at all occurs in the model. Further south, where more rainfall occurs during the monsoon season, VECTRI in general simulates somewhat too high *EIR*_*a*_ values in comparison to the rural observations. VECTRI also seems to simulate too high transmission values in equatorial Africa in Douala, which is subject to high annual rainfall rates. The simulated *EIR*_*a*_ values exceeding 500 infectious bites per human per year are rarely observed in Africa with 13 surveys out of a total of 180 reported by [32] registering *EIR* in this range.

**Figure 8:**
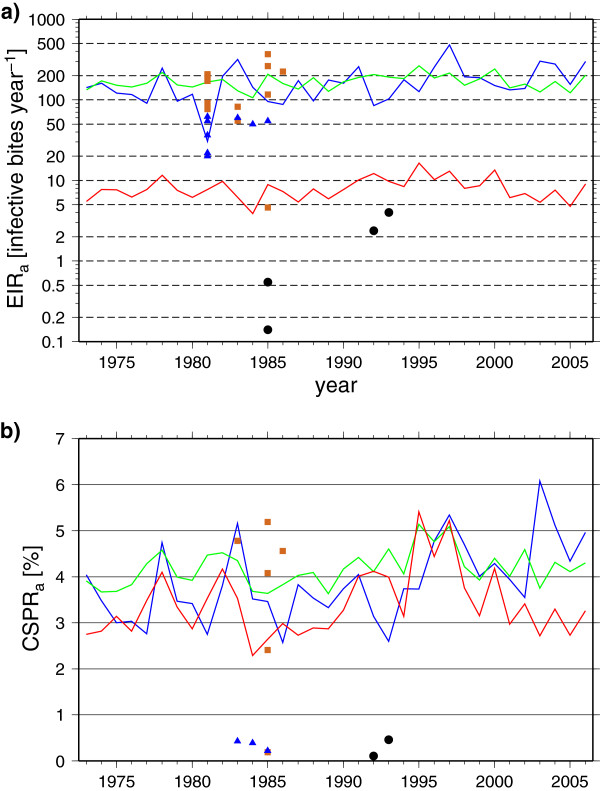
***EIR *and *CSPR *interannual variability in Bobo-Dioulasso.** Simulated and observed malaria transmission in the area of Bobo-Dioulasso. (**a**) Annual *EIR* rates (*EIR*_*a*_; in infectious bites per human per year) and (**b**) annual mean CSPR (*CSPR*_*a*_; in %). The blue lines represent LMM_2010_ and the red and green lines VECTRI simulations (red: human population density *H* = 1037 km^*-2*^; green*: H* = 32.2 km^-2^). The brown squares, blue triangles, and black dots stand for observed *EIR*_*a*_ and *CSPR*_*a*_ values in rural, irrigated and urban areas, respectively. See [30] for a definition of rural, irrigated and urban areas and for the sources of the field observations (their Additional file Two; the Word Meteorological Organisation (WMO) station number 65510 of Bobo-Dioulasso is assigned to the data).

A further detailed analysis is made for Bobo-Dioulasso in southwest Burkina Faso, which was chosen due to a particularly large number of field experiments with which to compare the models. Malaria is seasonally endemic in this location and a number of field campaigns have sampled *EIR* and *CSPR* in both (peri) urban and rural (some with nearby irrigation schemes) environments [97-106]. Only one integration is conducted for the LMM_2010_ since it is unable to account for population density. This is compared to two VECTRI integrations representative of rural (latitude 11.43°N, longitude 4.25°E, human population density *H*=32.2 km^-2^) and urban (latitude 11.2°N, longitude 4.30°E, *H* = 1040 km^-2^) field locations. The timeseries of annual *EIR* and *CSPR* values from the 3 integrations (Figure 8) show that both models reproduce the correct order of magnitude for these two variables in the rural location. The *EIR* in the VECTRI model and LMM_2010_ lie within the spread of measured *EIR*. The significant variability between the observed *EIR* values is noted, which could derive from differences in terrain, topography, altitude, vicinity to water bodies, irrigation and land use, interventions and simply experimental sampling error. It emphasizes the uncertainty in individual measurements and the need for ensembles of experiments to gauge this uncertainty. The *EIR* is generally lower in rural locations with irrigation, possibly as a result of preditor establishment in longer-lived pools but may also be due to irrigation providing farmers with higher incomes permitting further prevention and treatment measures. This emphasizes the difficulty in gauging such influences in the *w*_0_ parameter of the VECTRI model.

Intercomparing *EIR* from the two malaria models, the very high interannual variability of the LMM_2010_ is obvious, related to its elevated sensitivity to rainfall demonstrated in the earlier constant-input experiments. The LMM_2010_*EIR* value ranges by more than an order of magnitude between the lowest and highest year, while the interannual variability is less than a factor of two for the VECTRI model. It is interesting to note that, despite the basic underlying structure being very similar in the two models, the parametrization choices, in particular the implementation of the surface hydrology in the VECTRI model, results in almost a zero correlation between the two models in their representation of interannual variability; indeed, the *EIR* appear anti-correlated indicating that rainfall variability is determining the interannual variability to a large extent.

The black squares in the figure give *EIR* values for high population areas in peri-urban Bobo-Dioulasso, which are much smaller than in the rural environments but nevertheless non-zero. The LMM_2010_ is not designed to simulate these urban cells, however the VECTRI model is seen to reproduce reasonably well the contrast between rural and urban areas. Even though the treatment of the surface hydrology is identical in urban and rural environments - a gross oversimplification - the VECTRI model is able to mimic the drop in *EIR* which derives merely from the lower ratio of vector to host in urban areas. In contrast with the other West African locations, urban transmission is overestimated in this location. In the second panel, it is seen that while both models are again similar and perform well in reproducing the observed CSPR rates, the VECTRI model does less well in reproducing the distinction between rural and urban environments. Although the CSPR is lower in urban environments, it is still far larger than the observations in VECTRI. An possible implication is that vector lifetimes for urban areas are too long in the model.

**Figure 9:**
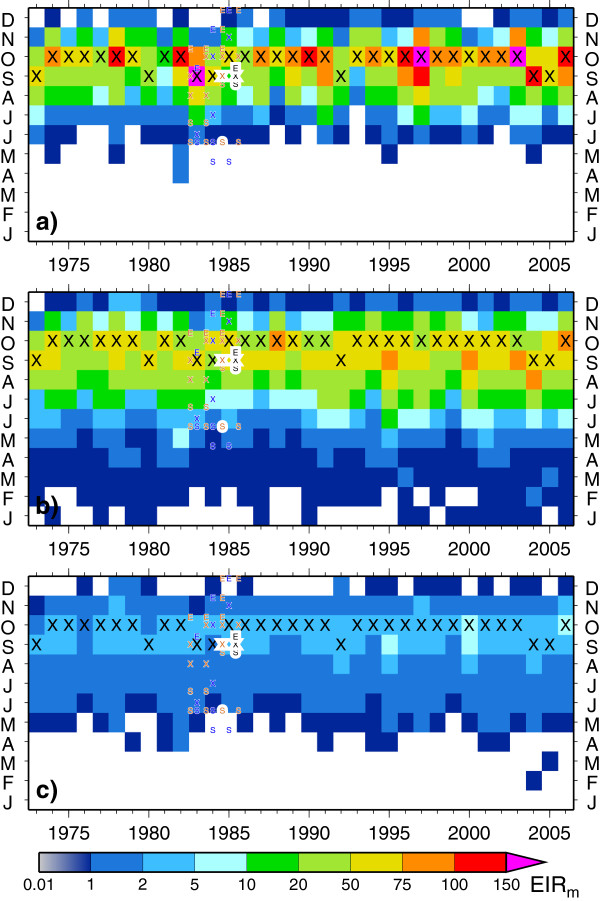
**Seasonal transmission in Bobo-Dioulasso.** Monthly mean *EIR* rates (*EIR*_*m*_) for (**a**) LMM_2010_ (**b**) rural VECTRI (density 32.2 km^*-2*^) and (c) urban VECTRI (density 1037 km^*-2*^). The large letter ‘X’ highlights the month in which the model produces a maximum in *EIR*. The start (‘S’), peak (‘X’) and end (‘E’) months for transmission measured in various field campaigns are marked in smaller font. The rural observations (brown) are placed on the left of the square of a particular month, the irrigated observations (blue) in the middle and the urban observations (black) to the right.

**Figure 10:**
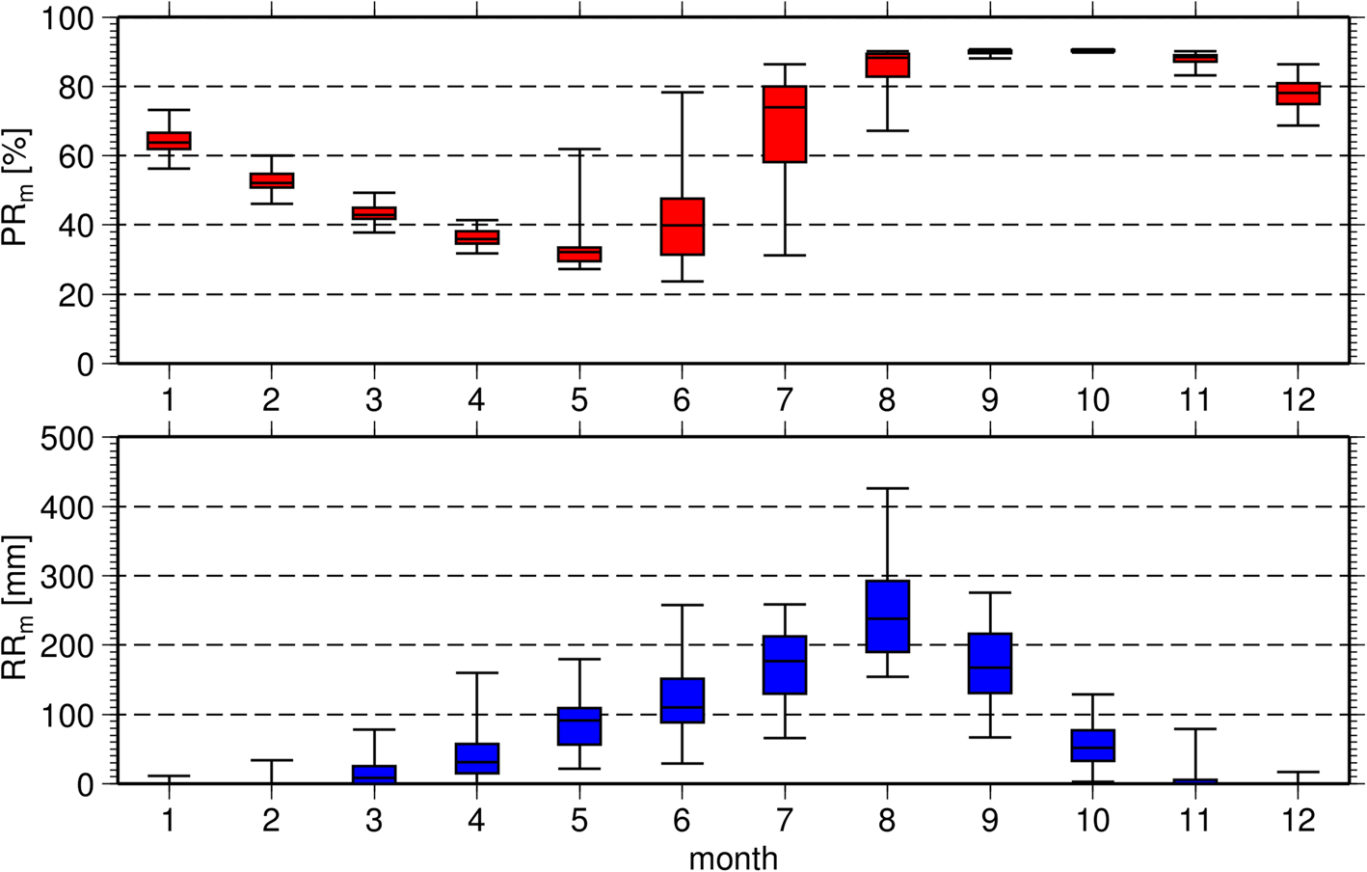
**Annual cycle of *PR *in Bobo-Dioulasso.** Annual cycle of monthly mean asexual parasite ratio (*PR*_*m*_; in %) for a rural area near Bobo-Dioulasso from the 34 year simulation of VECTRI. The boxes mark the 25 and 75% quantiles while the whiskers give the minimum and maximum values.

The seasonal cycle of *EIR* is shown in Figure 9 which demonstrates the onset, peak and consequential cessation of the season for the three model integrations, with symbols marking the same characteristics in observations. If transmission is assumed to be significant once the monthly *EIR* value exceeds 1, both models predict the timing of the onset, peak and cessation well for rural locations, although VECTRI maintains a very small background transmission rate for the two or three months prior to the onset. The higher interannual variability in the LMM_2010_ in the *EIR* value of the peak month is apparent. In urban environments the season is shorter, with a slightly later onset. It is notable that in 1982, both models predict an earlier than usual onset for the malaria season, while they disagree on an anomalous outbreak predicted by VECTRI in 2004. In fact, while health facilities in endemic areas are usually well prepared to deal with the regular transmission season, such information concerning the potential for an earlier than usual onset could be very useful in a forecasting system. This is also clear from Figure 10, which shows the PR annual cycle for rural location predicted by VECTRI. The box whiskers show the interannual variability, and it is seen that the greatest variability is in the onset phase of the malaria season. After the PR saturates in the peak of the season the interannual variability is limited. Thus predictions of an early onset could provide some valuable information to health planners even in endemic regions allowing them to better prepare for anonymously early seasons.

### Regional integrations

**Figure 11:**
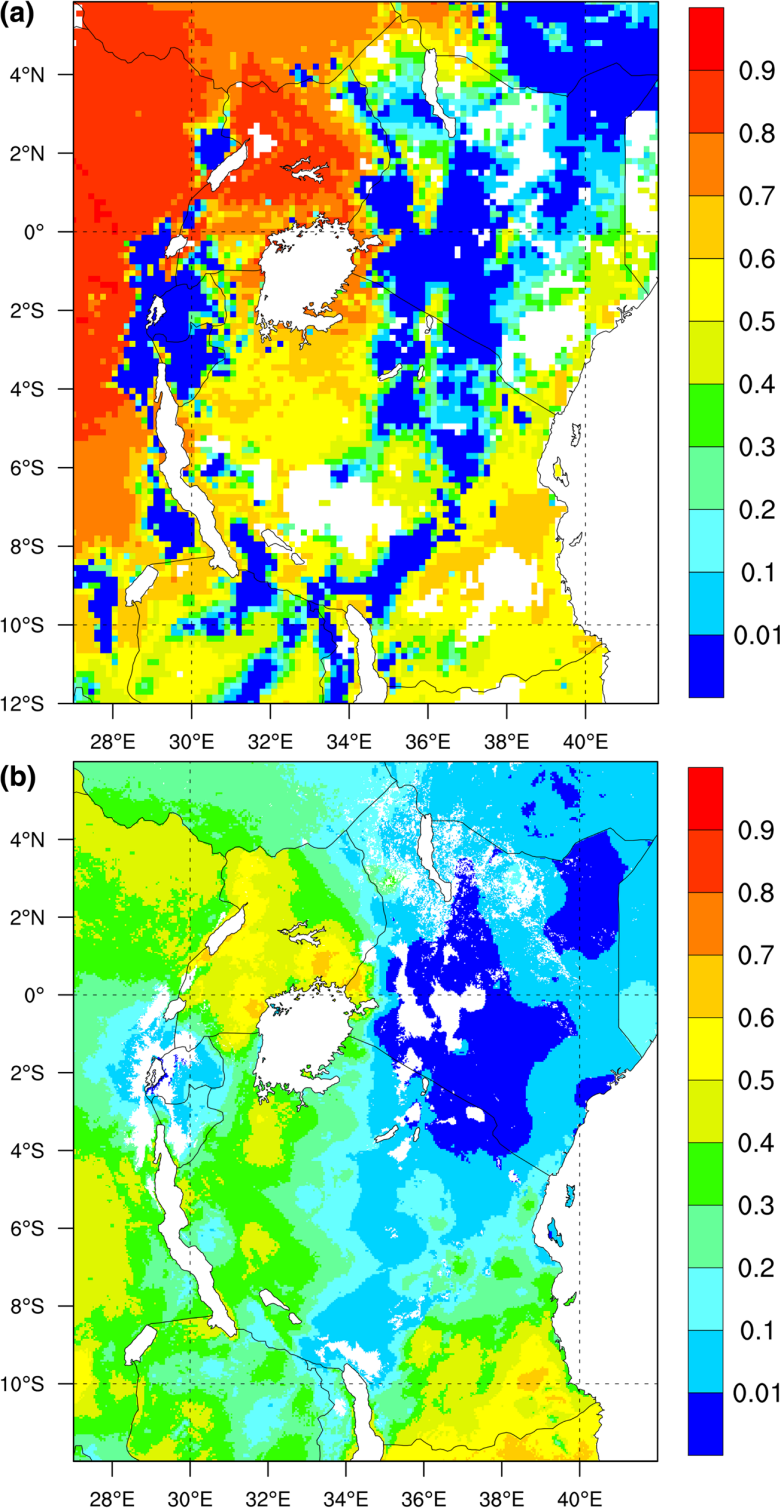
**Regional simulation of *PR *in Eastern Africa.** Mean asexual parasite ratio (PR; fraction) from (**a**) a 10 year-long VECTRI integration for East Africa and (**b**) MAP PR analysis. See text for details of model integration and data source.

The strength of the VECTRI model is its ability to run on a regional scale at a relatively high horizontal resolution. The VECTRI integration made for Eastern Africa is compared to the MAP malaria analysis (Figure 11). As this is a single deterministic integration of the malaria model it is simply compared to the mean PR of MAP, but future work will integrate the malaria model in a multi ensemble stochastic framework. This simple comparison of mean PR distributions indicates that the model is able to reproduce the general patterns observed in the MAP analysis, with high rates in central and northeastern Uganda and Western Kenya, but dropping over the higher terrain, with the central Kenya, central Tanzania and most of Rwanda largely malaria-free (*EIR*_*a*_ <0.01, *PR* <1%), as is the southwestern-most tip of Uganda.

The VECTRI model also reproduces the malaria zones in the warmer and more humid coastal regions in Kenya and Tanzania, but with values of PR along the Kenyan coast and northern Tanzania in VECTRI (30-70%) appear to greatly exceed those in the MAP analysis (0-30%). This is a region in which the survey data incorporated into the MAP analysis is dense, although Figure Three of [113] still identifies this coastal region as relatively uncertain. A large part of the discrepancy between VECTRI and MAP is likely due to the increasing interventions including widespread distribution of insecticide-treated nets (ITNs) that have occurred over the past decade that have greatly decreased parasite ratios and hospital admissions [117,118]. For example, the survey of 30 villages in Malindi, Kilifi, and Kwale Districts carried out in the late 1990s before ITN distribution started [119] (included in the MAP analysis) reported a parasite prevalence ranging from 38 to 83%, with a mean slightly exceeding 60% in each district, in close agreement with the VECTRI model. This highlights the importance of the future incorporations of interventions into VECTRI if it is to be applied to the seasonal forecasting task.

**Figure 12:**
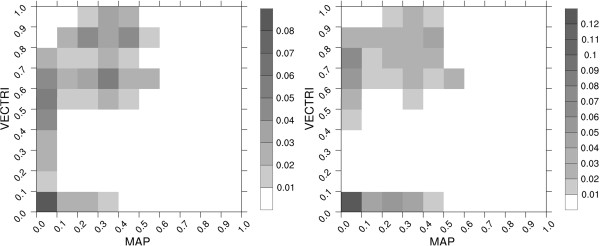
**Joint probability function of *PR.*** Joint probability density function of PR from MAP and VECTRI (**a**) with larvae growth rate fixed to 12 day cycle and (**b**) water temperature dependent growth rate according to [47].

One key new component of the VECTRI model is the potential for water temperature of the surface hydrology model to impact larvae growth rates over regional scales, however, it was highlighted that the relationship was highly uncertain. To quantify this relative sensitivity to the temperature of water, a second set of high resolution (0.1 degree, equivalent to approximately 11 km) regional simulations were repeated using a domain that covers both West and East Africa. The first uses the default fixed larvae lifecycle lifespan of 12 days while the second uses the degree day parametrization with degree day of 90 days. The VECTRI output is compared to the MAP data using a joint probability function (Figure 12). There is agreement on where regions are malaria-free, and a positive correlation between the MAP data and the two VECTRI experiments. In both cases the VECTRI model appears to overestimate the PR in locations where malaria exists compared to the MAP data, due to the lack of interventions and treatment in the VECTRI model. For example, the default clearance rate of 150 days is likely to be too long in endemic areas with widespread immunity. Moving from a constant larvae growth rate to a temperature sensitive growth rate sharpens the contrast in parasite loading between the lowlands and highlands, and this is seen in the disappearance of the intermediate values of PR in the VECTRI simulation, with PR either less than 0.1 or greater than 0.4 in these regions. This overestimation will likely improve once immunity and interventions are included in the model.

**Figure 13:**
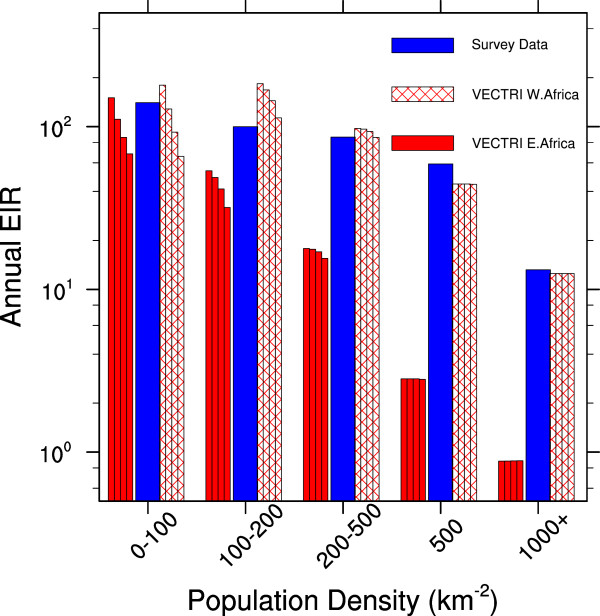
**Sensitivity of *EIR *to population density.** VECTRI modelled annual *EIR* as a function of population density divided into Western and Eastern zones of Africa, and the field survey data adapted from Figure Two of [33] (see their Figure One for survey locations). The VECTRI integrations were rerun with a range of values for τ_*zoo*_, shown by the thin bars, representing values of 35, 50 (default), 70 and 100 km^-1^ respectively from left to right.

One of the new elements of the VECTRI model is the incorporation of the population density in the calculation of biting rates and subsequent transmission probabilities. The decreasing biting rate as population increases has been previously noted by [31-33], although the relationships vary due to contrasting methodologies of calculating *EIR* and the differing locations of the studies. The sensitivity of *EIR* to population density in the deterministic East Africa integration (Figure 13) reveals a decreasing force of infection with increasing population. In Eastern Africa, the overall infectious biting rates are quite low in VECTRI, ranging from less than 1 year^-1^ in city year^-1^ in city centres to around 80-100 year^-1^ in rural areas, which is to be expected as much of the highlands are malaria marginal or free. In contrast the VECTRI *EIR* rates for West Africa, are greater, exceeding 150 year^-1^. The VECTRI West Africa domain excludes central Africa, for which still higher transmission rates are expected. The sensitivity of the *EIR* to population is comparable to the pan-continental field study summaries of [31], who reported a mean urban *EIR* of 7.1 year^-1^ increasing to 167 year^-1^ in rural areas. Likewise the analysis of 199 surveys by [33] reproduced in the figure lie in the range of the VECTRI simulations, although VECTRI appears to underestimate transmission at high population densities, relative to this particular study. Part of the reason for this could be the present neglect of population migration in VECTRI, which would tend to reduce the disparities between urban and rural *EIR* via its effect on also be the cause. For example, as mentioned above, the malaria clearance rates set to 150 days neglect the potential acceleration of clearance with strong immunity. The slow clearance leads to PR values that are either close to zero in malaria unsuitable zones, or between 0.4 and 0.9 in endemic zones. In addition, the survey data is subject to high uncertainty due to sampling, highlighted by the contrast between the results of [31,33] for urban areas.

It is emphasized that these are single deterministic integrations of the VECTRI model using its default parameter settings, and an important next step will be to document how sensitive this relationship is to the parameter settings in a stochastic integration framework [18]. Nevertheless, Figure 13 includes the sensitivity to the parameter τ_*zoo*_, chosen as it is a tuned parameter in the model, with integrations using values of 35, 70 and 100 km^-2^ also applied. As expected, these altered values impact the biting rate at low population densities, with lower values τ_*zoo*_ significantly increasing *EIR* in sparsely populated areas. Above, 200 people per km^-2^ the parameter setting has no impact, and for all settings the model over-sensitivity relative to [33] remains. This is because the parameter is designed to account for increasing zoophilicity at very low population densities, and thus are unable to represent the impact of human population movements which impact PR at low and high population densities. Nevertheless, while the model presently neglects differences in surface hydrology in peri-urban versus rural areas [120], urban micro meteorology and housing differences [121], the result indicates that a zero order impact of population density is the dilution effect, where increasing population densities reduce the probability of parasite transmission to the vector [31,122].

## Conclusions

A new open source community model (contact the corresponding author) for malaria is presented that specifically represents dynamical equations for the relevant temperature sensitive cycles in the transmission of malaria, namely the larvae growth cycle in addition to the sporogonic and gonotrophic cycles. Each cycle is divided into an array of bins to resolve the respective processes in order to model the delay in the malaria season with respect to the driving cycle of rainfall, while temperature affects development rates. The VECTRI model implements a framework to consider the surface hydrology of temporary water bodies and ponds. It also incorporates the human/vector element by accounting for population density in the calculation of transmission probabilities, enabling the VECTRI model to differentiate between urban, peri-urban and rural environments and permitting the future incorporation of various interventions into the modelling framework in addition to a treatment of urban-rural and transnational migration.

The model has been integrated in an idealized equilibrium environment to compare the sensitivity of weather parameters to an existing dynamical model LMM_2010_, and the contrasting response to rainfall due to the incorporation of surface hydrology in VECTRI was noted. Two further sets of experiments confronted the model with a range of observational data for infectious biting rates and parasite ratios in the human population taken from several hundred field studies mostly based in West or Eastern Africa. The model reproduces mean rural infectious bite rates for a range of locations throughout West Africa quite well, with a seasonal cycle of PR that appeared reasonable.

The incorporation of the population density in the biting rate calculation enables VECTRI to differentiate peri-urban and rural environments and it was demonstrated that the model was also able to reproduce the much lower *EIR* and CSPR rates measured in a peri-urban suburb environment of Bobo-Dioulasso less than 20 km distant from a rural site with far higher transmission. This is interesting as it indicates that simply representing the lower ratio of vector to human numbers is sufficient to represent the lower transmission intensities generally found in peri-urban environments without invoking differences in surface hydrology, access to health facilities, housing quality or use of ITNs. Regional integrations for Eastern Africa highlighted this ability of VECTRI to grossly reproduce *EIR* rates as a function of population density, in addition to its reasonable distribution of endemic and malaria free regions, separated by intermediate altitude, epidemic regions, although relative to the MAP data, the VECTRI model tends to overestimate PR in endemic zones. Whether this is due to missing processes, such as immunity or migration, or poor parameter choices remains unclear and the subject of investigation.

While already representing a useful tool to aid research into the understanding of malaria transmission, the VECTRI is undergoing further development to incorporate further aspects important for malaria transmission. These developments include: a flexible incorporation of various models for host immunity, a stochastic ensemble framework to account for uncertainty in model parameters and observations, improved surface hydrology that accounts for terrain slope, soil type and land use characteristics, a dynamical host model that includes migration, population demographics and urbanisation, explicit incorporation of commonly employed interventions, and lastly multiple vector types and vector dispersion.

## Competing interests

The authors declare that they have no competing interests.

## Authors’ contributions

AMT designed and developed the VECTRI model, performed the VECTRI integrations and drafted the manuscript. VE advised on model parameterization scheme choices in VECTRI, developed the West Africa station test suite and performed the LMM_2010_ integrations for the model intercomparison. Both authors read/approved and produced graphics for the final manuscript.
